# An Exploration of Complementary Feeding Practices, Information Needs and Sources

**DOI:** 10.3390/ijerph16224311

**Published:** 2019-11-06

**Authors:** Ada L. Garcia, Sarah Looby, Kimberley McLean-Guthrie, Alison Parrett

**Affiliations:** 1 Human Nutrition, School of Medicine, Dentistry and Nursing, College of Medical, Veterinary and Life Sciences, University of Glasgow, Glasgow G31 2ER, UK; Ada.Garcia@glasgow.ac.uk (A.L.G.); sarah.looby@ucdconnect.ie (S.L.); 2 Healthy Mummy Happy Baby, Lanarkshire Community Food and Health Partnership, Bargeddie G69 7TU, UK; kimberleyamclean@gmail.com

**Keywords:** weaning, complementary feeding, introduction of solids, UK weaning practices, infant nutrition, weaning information, parents information sources

## Abstract

Following complementary feeding (CF) guidelines might be challenging for mothers lacking time, resources and/or information. We aimed to explore CF practices, information needs and channels used to obtain information in parents living in areas of socioeconomic deprivation. Sixty-four parents of infants aged 4–12 months completed a short questionnaire and 21 were interviewed. Mean (SD) weaning age was 5 ± 2.5 months, foods given >7 times/week included commercial baby foods (33%) and fruits (39%) while 86% gave formula daily. The main sources of CF information were friends and family (91%), the internet (89%) and health visitors (77%). Online forums (20%), e.g., Facebook and Netmums, were used to talk to other parents because they felt that “not enough” information was given to them by health professionals. Parents felt access to practical information was limited and identified weaning classes or online video tutorials could help meet their needs. Themes identified in qualitative findings were (1) weaning practices (i.e., concerns with child’s eating; and (2) information sources and needs (i.e., trust in the National Health Service (NHS) as a reliable source, need for practical advice). In conclusion, parents are accessing information from a number of non-evidence-based sources and they express the need for more practical advice.

## 1. Introduction

The introduction of solid foods is an important milestone during the first year of development. Often referred to as complementary feeding (CF) or weaning, the giving of food in addition to breast milk usually occurs at around six months of age; as breastmilk is no longer sufficient to meet the infant’s nutritional needs. Current World Health Organisation (WHO) guidelines recommend delaying the introduction of solids until six months (WHO 2003), while continuing breast feeding. Other specific recommendations include providing iron-rich sources, avoiding sugar and salt, encouraging use of suitable home-made foods and delaying cow’s milk until one year of age [1]. 

In the UK, there is high awareness of the recommendation that babies should not be given solid foods before 6 months of age, but there is little understanding of why or how to follow recommendations; in particular aspects related to development of the infant’s digestive system [2], the newer evidence on risk of allergies [3] or the importance of development of food preferences and establishment of healthy eating habits. This coupled with confused messages from professional bodies [4] and an influx of information on baby food labels which do not follow WHO recommendations on the age of baby food introduction [5] represent a challenge for parents/carers and also, health professionals. 

The practice of early introduction of solids between 2005 and 2010 has declined in the UK [6]. However, a survey of infant feeding practices in Scotland, showed 96% of infants were at least 4 months old when complementary foods were introduced, an improvement from previous statistics; suggesting that there is a shift towards delaying weaning from before 4 months to 6 months [7]. This is a positive endorsement of the effectiveness of promoting correct infant feeding practices when health practitioners provide a uniform message. However, the survey also highlighted that not all infants are introduced to solid foods at the correct time, or with the quality of food recommended; and identified more at-risk groups. Older mothers (over 30 years old) were more likely to follow correct weaning practices compared with younger mothers (below 30 years old). Mothers living in the most deprived areas of Scotland were more likely to wean earlier, more likely to use commercial baby foods and less likely to give fruit and vegetables compared with mothers in the least-deprived areas [7].

It is argued that getting the right start to a baby’s first food experiences can be a time of stress for parents and caregivers [8]. So, it is not surprising that parents consider seeking out the correct CF information an important part of the infants’ first year. In a survey involving 2366 women reporting experiences during maternity care, 39% reported unmet information needs of post-natal care [9]. Information on CF was one of the top-three information needs identified by mothers, as they sought information on weaning age, snack choices, healthy eating and fruit and vegetable intake [8]. Places where mothers seek out information varies depending on frequency of use, importance and usefulness of the source. In the UK, health visitors (HVs) and general practitioners (GPs) are viewed by parents/carers as providing the right information but peer pressure and inconsistent advice from other sources can lead to mother’s confusion [8,10]. In the UK context, speaking to friends and family members (33%), websites (55%), health care professionals (55%), books and other printed materials (49%) were among the most popular sources of CF information identified [7,8]. Friends or family were more likely to be used by first-time mothers (46%) as a source of information [7] but advice from other mothers is also highly valued and actively sought [8]. The internet was a popular source, with 55% of mothers using the web to search for weaning information [7]. However, there is no evidence to inform what weaning advice mothers are being given by these sources, what their specific information sources are and how this knowledge shapes their weaning behaviour. Thus, the aim of this study was to explore CF practices of parents in North Lanarkshire, an area of high economic deprivation in the West of Scotland. We also sought to find out where parents sourced information on CF and what type of information they needed.

## 2. Materials and Methods 

### 2.1. Study Design and Sample Size

This was a cross-sectional study using a mixed-method approach to explore current weaning practices and information sources and needs of parents and caregivers in North Lanarkshire. The University of Glasgow College of Medical, Veterinary and Life Sciences Ethics Committee approved this study procedure for Non-Clinical Research Involving Human Subjects (Application Number. 200170130). Given the exploratory and qualitative nature of the study, we did not calculate a sample size but aimed to reach saturation in terms of qualitative findings through individual interviews.

### 2.2. Inclusion and Exclusion Criteria

Subjects were included in this study if they were the parent or full-time caregiver of an infant aged between 4 and 12 months. Participants had to be living within North Lanarkshire and have English as their first language. Parents and caregivers of infants under 4 months or over 12 months of age, not currently consuming any form of solid food or not living in North Lanarkshire were excluded. 

### 2.3. Recruitment

Participants were recruited between June and July 2018 by word of mouth and via advertisements placed in relevant public places such as baby and toddler playgroups, local libraries and local playgrounds and supermarkets. Online social networking sites such as Facebook and Twitter were also used for recruitment. Recruitment was also facilitated by Lanarkshire Community Food and Health Partnership (a third sector organisation working locally) and Public Health Improvement in North Lanarkshire Council, a government organisation. Prior to inclusion, all participants gave their written informed consent. 

### 2.4. Procedure

Participation involved the completion of either a questionnaire (paper copy or online), a structured interview, or both, as determined by the participants. Interviews were conducted in-person, however, if this was not possible, phone interviews were conducted. Subjects were informed that all responses would be anonymised and, if the subject consented, interviews were recorded. 

### 2.5. Materials

#### 2.5.1. Questionnaire

The questionnaire consisted of elements from a validated electronic survey which was designed for health staff assessment of feeding at 12 months in the West of Scotland context [11]. This questionnaire aimed to identify specific food groups which were consumed more or less frequently according to parents of 12 month old infants in the validation study. Such groups included green leafy vegetables and sour fruits, e.g., citrus fruits and green apples (less frequently) or fruit juice and sweet snacks (more frequently). In addition, we asked participants about their information-seeking behaviours and their experiences with weaning. We added specific questions on vitamin supplements upon request from the North Lanarkshire council that oversees the uptake of these supplements as part of policy recommendations. Participants were also asked demographic information including age, education level, postcode and number of children. The questionnaire is available as Supplementary Material.

#### 2.5.2. Interview

A structured interview was developed between the researchers and the stakeholders (Maternal and Infant Nutritionist form North Lanarkshire Council and Nutritionist from Lanarkshire Community Food and Health Partnership) in order to further investigate the various practices, perceptions, information-seeking behaviours and concerns of parents and caregivers in North Lanarkshire. The interviews were one-off and used a one-to-one format to collect qualitative data, either in person or over the phone. Interviews by a single interviewer followed a standard protocol [12]. Interviewees were asked to focus on their youngest child, if they had more than one, when discussing weaning. A pilot interview was conducted to test the suitability of the questions and to assess interview length. Each interview lasted around 10–15 min. The schedule of questions was as follows: 1. Can you talk us through the way you weaned/introduced solids to your baby? 2. How did you feel about the first experiences with weaning? 3. What are/were your concerns regarding weaning? 4. Where did you receive information regarding weaning? 5. What kind of information were you given? 6. Were you given any practical advice/support—elaborate? 7. What do you think would be/could have been useful to help you during weaning?

### 2.6. Data Analysis

For all statistical analysis, IBM SPSS 24 was used. Descriptive statistics were presented as percentages and means +/− standard deviations. Postcodes were used to evaluate deprivation according to the Scottish Index of Multiple Deprivation [13]. Quintiles 1–2 represent areas of high deprivation, and quintiles 3–5 areas of low deprivation. We did not carry out statistical analysis due to the small sample size and because the main outcomes of this study were based on qualitative findings.

The recorded interviews were transcribed and analysed using NVivo software version 12 (2018). The software helped to identify and code for common themes within the text. Key themes were highlighted according to both the prevalence within the text and the importance of the topic in relation to the research.

## 3. Results

### 3.1. Participant Characteristics

A total of 68 subjects (67 mothers, 1 father) participated in this study. All participants completed the questionnaire. Four participants were excluded from analysis because their infants were younger than four months or were not yet receiving solid foods at the time of data collection. Therefore, 64 subjects were included in the analysis and 21 participants consented to an interview. Participant characteristics are displayed in Table 1. Parental age was asked for both parents, mothers mean (SD) age was 30 (6) and fathers 33 (6) years, the minimum age was 20 and maximum 57 years. The majority of parents lived in socioeconomically deprived households (52%) but reported a medium to high level of education (90%). The mean (SD) age of infants was 7.5 (2.5) months, minimum age of 4 and maximum 12 months. The sex of the infants was almost equal (53% male vs. 47% female).

### 3.2. Current Feeding Practices in North Lanarkshire

#### 3.2.1. Age Introduced to Solids

The mean (SD) weaning age in this sample group was 5 (2.5) months with a minimum and maximum of 3–6 months. The majority (97%) of infants were weaned after 4 months, while just 34% were weaned according to current recommendations at 6 months of age. Most parents reported that their infant was breast-fed (61%), but the mean (SD) duration of breast feeding was only 2.7 (3.4) months. Those living in the most-deprived areas were more likely to introduce solid foods earlier, ≤4 months (20%) compared with participants who lived in the least-deprived areas (8%).

#### 3.2.2. Types of Food and Drinks Given

The most common foods given to infants were commercial baby foods, other fruits (such as bananas and apples), and green leafy vegetables, with around 1/3 of infants receiving them 7+ times per week (Table 2). These foods were the key components of the infants’ diet in this sample. Sweet snacks, such as cakes and biscuits, sour fruits, such as orange, and meat, fish and eggs, were least commonly given to infants with 69%, 50% and 44% (respectively) consuming them never or just once per week.

The most popular drinks infants received, at least once a day, were infant formula (86%) followed by water (80%) (Table 2). Nine of the 64 infants included in the study were still being breast-fed at the time of data collection. Some participants reported giving drinks that are not recommended for infants, such as cow’s milk (6%) and diluting juice (17%), at least once per day

There were differences in diet depending on whether participants lived in the most/least deprived areas of North Lanarkshire. Participants living in areas of high deprivation were much less likely to give their infants sour fruit compared with those in areas of low deprivation (48% vs. 97%), and less likely to give other fruits (27% vs. 30%) and green leafy vegetables at least once a week (16% vs. 22%). Participants living in areas of low deprivation were more likely to give their infants meat/fish/eggs at least once a week compared with those living in areas of high deprivation (77% vs. 48%). Prevalence of commercial infant food (77%) and sweet snacks (45%) use was higher among those living in areas of low deprivation compared with more deprived areas (67% and 30%, respectively).

#### 3.2.3. Feeding Methods

Infants commonly received three meals and snacks per day (36%) although 30% of participants gave more than three meals plus snacks a day with 18% receiving just one meal/snack a day, this depended very much on the age of the baby. Giving finger foods was more popular than spoon feeding, with 69% of infants feeding themselves finger foods at least once per day vs. 41% who spoon-fed themselves. Participants experienced very little difficulty when it came to feeding their infants, with only 7.8% reporting any feeding difficulties and a parental reported appetite score of 4.8 (0.9) (mean (SD)) where 1 = very poor to 6 = exceptionally good. However, it was much more common for participants to report that their infant was a ‘fussy eater’, 50% found there were tastes, textures, flavours or foods their infant did not like.

#### 3.2.4. Vitamin Supplements

Overall, vitamin supplement uptake was low among participants, with only 25% of parents giving their infants some form of vitamin supplement. The awareness of the vitamin recommendations for infants was low, with only 31% of participants reporting to know the recommendations. Of those who were aware, 50% had received the information on vitamin recommendations from a health visitor, 15% had found it on the NHS website, 15% from a weaning class and 10% from other internet sources.

### 3.3. Concerns of Parents in North Lanarkshire

Allergies (70%), choking (66%) and knowing what foods to give (64%) were the top three concerns among participants. Other concerns among participants included salt content of foods and baby-led weaning. Participants were least concerned about knowing what is healthy (28%), giving sweet treats (19%) and organic foods (11%).

### 3.4. Information Sources and Needs of Parents in North Lanarkshire

When asked how much information (none, some, a lot) they received on weaning, the majority (61%) of parents in North Lanarkshire reported that they received some information. The rest of participants were divided between the extremes, either receiving a lot of information (11%) or not enough (24%).

**Figure 1:**
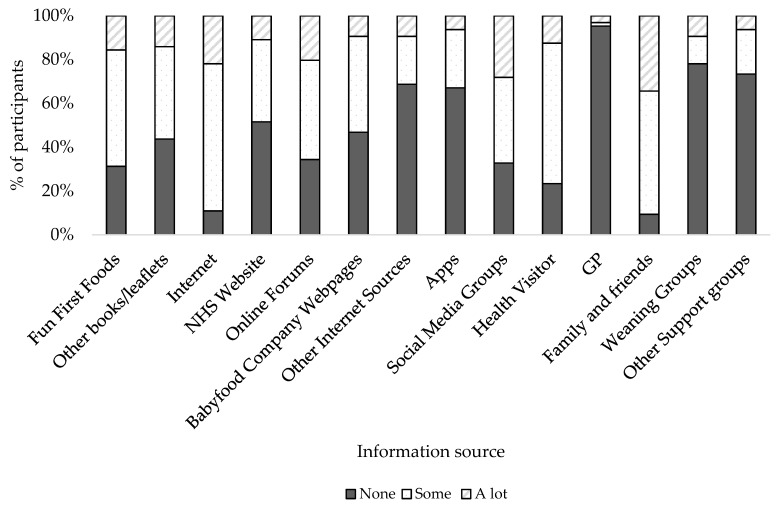
Percentage (%) of parents reporting the amount of information (none, some, a lot) they received from each information source.

Participants received a lot of information from family and friends (34%), social media groups (28%) and the internet in general (22%) but, particularly, from online forums (20%) (Figure 1). Participants reported receiving some information from a variety of sources including the internet (67%), Health Visitor (64%), family and friends (56%) and the Fun First Foods booklet from the NHS (53%). Other internet sources participants used for some information include baby food websites (44%) and the NHS website (38%). Weaning groups, other support groups and general practitioners were the least utilised resource, with the majority of participants receiving no advice from these sources (73%, 78% and 95%, respectively) (Figure 1). Apps provided participants with some information (27%) and frequently used apps included Bounty and apps created by baby food companies, Ella’s Kitchen and Cow and Gate.

In general, the kind of information participants reported receiving from these sources were recommended weaning age (95%) and the types of food to give (89%) and to avoid (75%). It was less frequent for participants to receive practical advice such as how to cook (48%) and store baby food (38%), nutrient information (47%) or the economic benefits of preparing home-made foods.

#### Information Needs

A few participants (5%) reported not to have received information on complementary feeding. These participants, along with those who felt they hadn’t received “enough” information, reported that different ideas for menu plans, portion-size recommendations and more information on baby-led weaning was the kind of information they specifically would have liked to have received. The rest of the participants indicated that their need for information was more related to how and where to access information (sources) as follows: small group sessions with demonstrations (73%), videos with ideas of portion sizes (69%) and menus (67%) and apps (66%). Videos were generally considered to be a useful idea, in particular, those offering portion-size examples (69%) and menu ideas (67%), followed by videos on the types of baby food to give (59%), how to cook baby food (52%) and how to feed an infant (47%). Other suggestions (3%) included recipe booklets and additional information on nutritional requirements, specifically salt and sugar, and calorie intake. Weaning Fayres, an open day session to discuss weaning delivered by health practitioners in the West of Scotland, (11%) were not considered useful for advice about weaning.

### 3.5. Qualitative Data

After transcription, common themes were highlighted according to both the prevalence within the text and the importance of the topic in relation to the research. Themes were divided into two categories: (1) Weaning practices: expectation vs. reality; mothers’ concerns with child’s eating and (2) Information sources and needs: the NHS; practical advice; need for more.

#### 3.5.1. Weaning Practices

##### Expectation vs. Reality

A common theme expressed in the interviews was that mothers often had an expectation of what weaning would be like and how they would do things but in reality, things did not go as planned.

When talking about how they felt mothers commonly expressed a level of excitement at the thought of weaning, before they actually started. However, they went on to report that, once they began to feed infants’ solids, it was not what they expected.

*“at first I was really excited, and I couldn’t wait to start and sort of couldn’t wait to start him on food, he’s going love it, it’s going to be great then obviously it was really, quite a bit disheartening because he wasn’t that interested”*.

(P08)

The feelings of excitement and eagerness to start weaning turned to slight disappointment and apprehension when mothers discovered things were not going to be as simple as they had originally thought.

*“I was really excited, I love it, I love watching them try new things and see how they react. But then when you have to introduce more lumps I get apprehensive, like will he choke and all those sorts of things”*.

(P24)

When it came to the guidelines, many mothers were aware of the recommended starting age for weaning, expressing an intention to wait until six months before introducing solids. However, a number of mothers chose not to follow them, instead opting to wean earlier.

*“I wanted to wean him early… I know the guidelines is six months”* (P07), or *“just felt like [the infant] was ready”.*

This was not the case for all participants, one mother in particular, saw no reason to start before the current recommendations:

*“I followed them, she was fine with her milk, she was satisfied with her milk, I didn’t feel any reason to go before the recommendations”*.

(P20)

Mothers who did wait until 6 months often reported that this was due to the advice of a health professional.

When mothers were prompted on why they started weaning when they did, particularly if they admitted to starting before 6 months, it was common for mothers to list hunger and an interest in food as reasons. In terms of hunger, mothers often reported that infants started consuming more milk and so took this as a sign the infant was ready for more food in the form of solids

*“he was guzzling milk, he was up to 32oz a day”*.

(P25)

Infants expressing an interest in food by grabbing at it or watching others eat was another sign that convinced mothers their infant was ready to start weaning

*“she was constantly grabbing food off of everybody else, so it was like you may as well have, you may as well start having some food.”*

(P04)

##### Mothers Concerns with Child’s Eating

When it came to weaning styles, mothers’ opinions suggest that “trying finger foods” could have been exciting, probably because it shows a developmental milestone, but this excitement turned to concern. Mothers seemed less confident introducing finger foods compared with purees, often starting with baby rice, porridge or purees to build up confidence before progressing on to finger foods or baby led weaning (BLW).

*“until I kind of got more confident and then I started the baby-led weaning”*.

(P42)

Participants often expressed a desire to try or attempt to progress to BLW as though they considered it the correct method of weaning. Concern or lack of confidence deterred many of the mothers from starting with BLW or trying it at all. This often arose from the fear of the infant choking, specifically in relation to finger foods and the methods of BLW

*“I was debating whether I was going to let her do it herself, baby-led weaning, and backed out of it because I was petrified of her choking”*.

(P18)

Allergies also worried mothers. Thematic analysis highlighted the different ways mothers in NL were choosing to manage their concerns in relation to how they fed their infants. There was a divide among participants who either avoided allergens completely when weaning

*“You don’t really get information on what if they do have a severe allergy. So I’ve tried to keep the allergy foods to a minimum”*,

(P18)

or who slowly introduced them one-by-one, to ensure the infant had no reaction

*“I introduced the same foods for a number of days to make sure there was no allergies there, especially things like strawberries and things that people are prone to have allergies”*.

(P07)

Five mothers opted to introduce allergens early, meaning they introduced them along with first foods during weaning to “get them out of the way”. While two mothers chose to completely avoid allergens, due to a family history of allergies, so they felt their child was more at risk.

Mothers reported a need for more information on portion sizes for infants. They expressed a lot of uncertainty over knowing how much to feed their infant leading to a concern that they weren’t feeding them enough

*“Portion size and things like that I was not really too sure what to give”*.

(P20)

Two mothers were more concerned that they were feeding their infant too much food. One mother talked about a fear of making her child obese later in life, which again stemmed from an uncertainty of knowing how much food to give.

#### 3.5.2. Information Sources and Needs

##### National Health Service (NHS)—The Official Advice

The NHS or information provided by the NHS was highly regarded by participants. In the UK, this is considered official governmental advice and, in the context of weaning, it is primarily delivered by HV’s. In Scotland, HV provide parents with the First Fun Foods booklet for complementary feeding guidance. In some areas, there are also ‘weaning fayres’ organized by NHS local teams, parents are invited to this ‘one off’ activity by their HV.

*“I did find the NHS book quite helpful just knowing what to give, what not to give”*.

(P06)

In particular, the NHS website was seen as a valuable, up-to-date resource for parents regarding weaning. It was noted by some participants that information surrounding weaning changes rapidly, that recommendations can change even between pregnancies and some felt that their HVs were not as up to date with these changes as the NHS webpage, and so utilised this resource instead

*“The NHS website is very up to date, but I just think there’s a wee bit of, maybe the people who are out in the community do need to catch up with the current information a wee bit because it doesn’t seem to marry up.”*

(P17)

The visit from the HV and receiving the NHS Fun First Foods booklet was usually mentioned when discussing where information came from. There was an element of dissatisfaction among the sample group when it came to the advice from the HV and the NHS booklet. Mothers described their talks with the HV as simplistic. They usually felt there was *“no other guidance”* given to them other than the basics or that the information they received was not what they wanted to hear. So, they put it aside and went in search of their own information. There was also a sense of mothers being too afraid to ask their HV questions when the information was not offered to them

*“often I don’t feel like I want to phone the health visitor up just to ask about something random”*.

(P06)

##### Practical Advice

There was a lack of awareness of weaning classes in local areas.

*“not that there was any class that I really saw that was available to be honest, because I maybe would have considered it if there was”*.

(P03)

A few mothers ( n= 3) who knew about and attended weaning classes in some form had received more information on practical elements such as the difference between commercial foods and home-made foods and what sort of foods they should be giving infants. Thus, better advertisement/signposting of these classes was suggested by one mother, who had attended a class where she was the only parent present.

Online videos were highlighted as a way of getting more practical advice out to parents, possibly attached to the NHS website or another legitimate, trusted source.

*“Online videos would be really, really good. I think a lot of the time videos are good, rather than recipes.”*

(P16)

These were seen as an alternative to classes and would be more accessible to people. Two mothers had already reported using YouTube videos to guide them on how to freeze and store baby food and how to feed their infant. Classes offer great practical learning opportunities but often it is more difficult for parents who have more than one child to find the time to attend them, so videos were a welcome alternative.

##### Need for More Information

It was common for mothers to report that they often used information from online sources. They turned to the internet because they felt they did not receive enough guidance from the HV. In particular, they reached out to other mothers or friends online through various forums and Facebook groups. A popular local Facebook group in NL was the Daisy Foundation (this is a local group that required payment for joining workshops), two mothers mentioned specifically using it to talk to other mums in the area.

*“it’s a really big Facebook page, it’s got 2500 members and it’s all local mums that’s on it.”*

(P07)

Mothers reported that they shared tips and advice with others via these groups. If they had any questions about weaning, or particularly, if they had a child with dietary restrictions, mothers would turn to these groups to see what others had done. It was seen as a much easier, faster method of getting advice rather than waiting for a health professional or HV to get back to them.

*“It’s easier to access as well, its quicker to access information online than it is to contact a health professional about it and wait until they get back to you”*.

(P17)

The structured interviews revealed that one of the main things mothers went in search of were different recipes and menu ideas. Some mothers struggled to come up with different menu options to give their infant to ensure they received a variety of foods. This need for different recipes seems to link to the mother’s uncertainty of what, when and how much, foods they can give their infants, so they look to see what other mothers have done.

*“it was more inspiration around meals, what they should be getting, initial foods that you should introduce them to, to begin with and they how to kind of build that in”*.

(P26)

## 4. Discussion

This study aimed to investigate current weaning practices and information needs of parents living in areas of social deprivation in the West of Scotland. Our findings show that parents in NL had introduced foods earlier than the recommended 6 months, fed infants a diet of commercial foods, fruits and vegetables, and rarely used vitamin and mineral supplements. Parents also had very specific information needs that are currently being meet through non-evidence-based sources, such as online outlets. Parents provided a series of suggestions that can be used to inform the development of resources to complement current advice given by official sources like the NHS.

Although we found a positive shift toward delaying the introduction of solids, deprivation still has an impact on the age of solid food introduction, parents living in the most-deprived households are more likely to introduce foods earlier or around 4 months of age as reported by the Infant Feeding Survey [7]. Our qualitative findings confirm previous reports that mothers respond to hunger cues and their infant showing interest in food as reasons to start solids [14] This implies further efforts are needed to highlight that these are not reasons to start weaning and to promote current feeding recommendations.

Perception of feeding difficulties among participants was much lower than previously reported (7.8% our study vs. 11% a wider UK infant feeding survey) [6] and, overall, parents reported their children to have a very good appetite, which might be expected in a healthy infant population. In our study, reports of “fussy eating” were much higher (50% our study vs. 4% in UK). This might be due to the lack of a clear definition of “fussy eating”, which might be open to misinterpretation [15]. Fussy eating is generally reported at later ages [14], thus it is a concern that parents are reporting fussy eating during CF. It has been shown that, on average, parents only offer an infant a new food up to five times, far fewer than the recommended 8–10 times, before deciding the child does not like it [16]. Recommendations from the SACN report have highlighted the need to present a new food to an infant a number of times before it is accepted [1], indicating the importance of encouraging parents to keep trying with new foods. Despite reports of “fussy eating”, overall vegetable consumption was high in our sample; however, we didn’t explore the diversity or variety of offered vegetables. It has been found that parents tend to only offer three different vegetables to infants in the first month of weaning [17]. From the qualitative findings on weaning practices, parents reported that expectations and reality did not always match. This led to parental concerns such as that introducing foods could lead to allergies and choking. In the case of allergies, there was “confusion” over the correct action to take. Other studies have shown that parents are still cautious about offering allergen-rich foods such as peanuts, with 8% of infants in the UK having consumed peanuts during weaning and only 5% in Scotland [6]. In terms of other allergens, 27% of infants in Scotland never received egg [7]. Previously, parents were advised to avoid allergens until 12 months of age; however, recent recommendations suggest that allergens should be a part of an infant’s usual diet when foods are introduced at around 6 months of age [1]. However, our findings suggest that the updated guidelines have not been clearly communicated to parents.

A further concern of parents in this study was choking when offering finger foods as part of BLW. This feeding style has grown in popularity over the last 10–15 years [18]. BLW advocates bypassing purées and allowing infants to self-feed solid foods from the start of weaning [19]. It has been associated with the later introduction of solids and reduced maternal stress during the solid-food introduction phase [20]. Evidence from our study suggests that mothers were keen to try BLW, but that the majority were apprehensive about introducing finger foods straight away, due to the risk of choking; and often started with purees to build confidence. Mothers also noted that HVs were less supportive of the BLW approach. Two studies investigating the attitudes of health professionals and mothers towards BLW, found that healthcare professionals had limited experience with it and expressed concerns about the potential increased risk of choking [21]. Mothers who followed BLW either had no major concerns [21] or were concerned about choking in the beginning before becoming more confident as weaning progressed [19]. In addition, due to the lack of convincing and robust evidence of the benefits/effectiveness of the BLW approach, it is not surprising that HV have a more evidence-based approach to recommending this weaning style.

Due to the limited evidence on parental information needs on CF, we explored the types of information being received from various sources and include some that are not evidence-based. Results show family and friends; the internet and the HV were the most-used information sources. This suggests an increased use of informal information sources in recent years. In 2010, only 29% of mothers reported using the internet in the UK, compared with 65% getting formal information from the HV [6]. In Scotland in 2016, 55% of mothers reported accessing information online, and results from this study show this has increased to 89% [7]. Interestingly, the BLW trend has been associated with seeking advice from the internet [20], most likely because parents/caregivers are not getting it from healthcare professionals due to lack of robust evidence [21]. Digital technology such as apps, social media and interactive websites, have the potential to improve population health by supporting behaviours involved in disease prevention and delivering evidence-based advice [22]. However, it may also prove harmful if the information offered is incorrect or non-factual.

Further insight into the types of websites parents access online showed that online forums and social media groups were frequently used as a means to talk with other parents. Internet forums provide UK mothers with valuable support on a wide range of parenting issues [23]. The common experience of other mothers is highly valued, especially by new mothers [8]. Parents in NL went online because they felt they needed very specific information. Nevertheless, parents in our study also said that they checked the NHS website for more up-to-date information.

From the data, the main types of information parents from NL are receiving include the age of introduction to solids and the types of food to give and avoid, these findings are similar to UK and Scottish statistics [7]. However, information gaps were identified. One specific information need highlighted in this study, was the need for more practical advice, delivered in the form of weaning classes or via online videos. Practical needs varied from how to cook and store infant foods, to ideas for menus and recipes. Practical advice on snack choice, healthy eating and including fruit and vegetables in the diet were needs also identified in another UK study [8].

Results highlighted a lack of awareness, time and opportunity, as potential barriers to accessing this type of practical weaning information. A previous study [10] had also highlighted these as information barriers. These barriers could be overcome in NL by advertising/signposting more mothers to ongoing classes in the community. A potential way of doing this could be through the HV, as they have direct access to all mothers.

The strengths of the study were accessing a large number of participants from a socioeconomic deprived background who were interviewed for the qualitative aspect of the study. This allowed an in-depth assessment of parental information needs. We also worked in collaboration with local health practitioners who provided us with a normative need that can be used for tailoring future programmes.

The limitations of the study lie in the observational and exploratory nature with a limited sample size for the quantitative data. Additionally, the interpretation of qualitative data is susceptible to interpretation bias by the analysist. The self-reported data of the questionnaire could be subject to recall bias, in particular, for those questions related to when foods were introduced and breastfeeding duration, the age range of 4–12 months was selected because this is known as the complementary feeding period. The cross-sectional nature of the data and attribution bias by the parent are further limitations.

The sample size failed to include both very young parents (<20 years old) and parents from lower educational backgrounds; therefore, results cannot be generalised to a wider population. We did not compare parents with one vs. those with more children, this should be considered in future studies because milk feeding experiences with the first child have been associated with recurrent milk feeding experiences in the second child [24]. Sample size for the quantitative aspect of this study was too small to draw any statistical significance, so results should be used only to provide insights into weaning practices in NL.

## 5. Conclusions

Weaning trends within NL are similar to that of the rest of Scotland. However, socioeconomic deprivation does play a role in health-seeking behaviours of parents and caregivers, the significance of which needs to be further explored in a larger study to find effect sizes. Parents are accessing information from a variety of sources, many of which involve communicating with other parents online. The type of information they are accessing includes information on age of introduction and types of foods to give or avoid. It is important that stakeholders are aware of parental information needs and how they currently meet these needs. These findings give real insight in to CF practices in NL which can help inform, plan and deliver, tailored CF interventions, within the local community, using the information sources parents are accessing.

## Figures and Tables

**Table 1:** Participant characteristics. Percentage (%) characteristics of the parents (n = 64).

Parents	n (%)
SIMD	
Quintile 1	16 (25)
Quintile 2	17 (27)
Quintile 3	12 (19)
Quintile 4	11 (17)
Quintile 5	8 (13)
First-time parent (% yes)	40 (63)
*Mother*	
Education level *	
Low	4 (6)
Medium	16 (25)
High	42 (66)
*Father*	
Education level **	
Low	9 (14)
Medium	21 (33)
High	24 (38)

* Low (no formal education or just primary), Medium (Scottish Standard Grade and Highers—Secondary educations), High—Degree or higher educations, n = 62 provided answers for maternal education. ** n = 57 for paternal education.

**Table 2:** Type of foods and drinks given to infants. Food data are frequency (n) and percentage (%) of offered food groups in number of times per week. Drink data are frequency (n) and percentage (%) of offered drinks in number of times per day (n = 64).

Times/Week	Meat/Fish/Eggs	Commercial Baby Food	Green Leafy Vegetables	Sour Fruit	Other Fruit	Sweet Snacks
**0**	24 (38%)	18 (28%)	10 (15%)	26 (41%)	7 (11%)	41 (64%)
**1**	4 (6%)	4 (6%)	3 (5%)	6 (9%)	2 (3%)	3 (5%)
**2–4**	14 (22%)	14 (22%)	20 (31%)	20 (31%)	19 (30%)	13 (20%)
**5–6**	7 (11%)	7 (11%)	8 (13%)	3 (5%)	12 (19%)	4 (6%)
**7+**	15 (23%)	21 (33%)	23 (36%)	9 (14%)	24 (37%)	3 (5%)
	**Breast Milk**	**Formula Milk**	**Cow’s Milk**	**Diluting Juice**	**Water**	**Other**
**0**	55 (86%)	9 (14%)	60 (94%)	53 (83%)	13 (20%)	63 (98%)
**1–2**	2 (3%)	7 (11%)	3 (5%)	8 (13%)	25 (39%)	1 (2%)
**3–4**	0 (0%)	29 (45%)	1 (1%)	1 (1%)	21 (33%)	0 (0%)
**5+**	7 (11%)	19 (30%)	0 (0%)	2 (3%)	5 (8%)	0 (0%)

## Supplementary Materials

The following are available online at https://www.mdpi.com/1660-4601/16/22/4311/s1: Questionnaire S1: An Exploration of Complementary Feeding Practices, Information Needs and Sources in North Lanarkshire.

## References

[1] Scientific Advisory Committee on Nutrition (2018). Feeding in the First Year of Life.

[2] Fewtrell M., Bronsky J., Campoy C., Domellof M., Embleton N., Fidler Mis N., Hojsak I., Hulst J.M., Indrio F., Lapillonne A. (2017). Complementary Feeding: A Position Paper by the European Society for Paediatric Gastroenterology, Hepatology, and Nutrition (ESPGHAN) Committee on Nutrition. J. Pediatr. Gastroenterol. Nutr..

[3] Muraro A., Halken S., Arshad S.H., Beyer K., Dubois A.E., Du Toit G., Eigenmann P.A., Grimshaw K.E., Hoest A., Lack G. (2014). EAACI food allergy and anaphylaxis guidelines. Primary prevention of food allergy. Allergy.

[4] Agostoni C., Decsi T., Fewtrell M., Goulet O., Kolacek S., Koletzko B., Michaelsen K.F., Moreno L., Puntis J., Rigo J. (2008). Complementary feeding: A commentary by the ESPGHAN Committee on Nutrition. J. Pediatr. Gastroenterol. Nutr..

[5] Garcia A.L., Raza S., Parrett A., Wright C.M. (2013). Nutritional content of infant commercial weaning foods in the UK. Arch. Dis. Child..

[6] McAndrew F., Thompson J., Fellows L., Large A., Speed M., Renfrew M. (2012). Infant Feeding Survey 2010.

[7] The Scottish Government (2018). Scottish Maternal and Infant Nutrition Survey 2017.

[8] Loudon K., Buchanan S., Ruthven I. (2016). The everyday life information seeking behaviours of first-time mothers. J. Doc..

[9] The Scottish Government (2014). Having a Baby in Scotland 2013: Women’s Experiences of Maternity Care.

[10] Harrison M., Brodribb W., Hepworth J. (2017). A qualitative systematic review of maternal infant feeding practices in transitioning from milk feeds to family foods. Matern. Child Nutr..

[11] Tully L., Wright C.M., McCormick D., Garcia A.L. (2019). Assessing the Potential for Integrating Routine Data Collection on Complementary Feeding to Child Health Visits: A Mixed-Methods Study. Int. J. Environ. Res. Public Health.

[12] Bryman A. (2008). Social Research Methods.

[13] The Scottish Government (2016). SIMD ranks and domain ranks. The Scottish Indicator of Multiple Deprivation.

[14] Doub A.E., Moding K.J., Stifter C.A. (2015). Infant and maternal predictors of early life feeding decisions. The timing of solid food introduction. Appetite.

[15] Taylor C.M., Wernimont S.M., Northstone K., Emmett P.M. (2015). Picky/fussy eating in children: Review of definitions, assessment, prevalence and dietary intakes. Appetite.

[16] Nicklaus S. (2011). Children’s acceptance of new foods at weaning. Role of practices of weaning and of food sensory properties. Appetite.

[17] Caton S.J., Blundell P., Ahern S.M., Nekitsing C., Olsen A., Moller P., Hausner H., Remy E., Nicklaus S., Chabanet C. (2014). Learning to eat vegetables in early life: The role of timing, age and individual eating traits. PLoS ONE.

[18] Brown A., Jones S.W., Rowan H. (2017). Baby-Led Weaning: The Evidence to Date. Curr. Nutr. Rep..

[19] Brown A., Lee M. (2013). An exploration of experiences of mothers following a baby-led weaning style: Developmental readiness for complementary foods. Matern. Child Nutr..

[20] Brown A., Lee M. (2011). A descriptive study investigating the use and nature of baby-led weaning in a UK sample of mothers. Matern. Child Nutr..

[21] Cameron S.L., Heath A.L., Taylor R.W. (2012). Healthcare professionals’ and mothers’ knowledge of, attitudes to and experiences with, Baby-Led Weaning: A content analysis study. BMJ Open.

[22] Michie S., Yardley L., West R., Patrick K., Greaves F. (2017). Developing and Evaluating Digital Interventions to Promote Behavior Change in Health and Health Care: Recommendations Resulting From an International Workshop. J. Med. Internet Res..

[23] Russell S. (2006). Netmums: Online support for parents. Community Pract. J. Community Pract. Health Visit. Assoc..

[24] Bentley J.P., Bond D., de Vroome M., Yip E., Nassar N. (2016). Factors Associated with Recurrent Infant Feeding Practices in Subsequent Births. J. Hum. Lact. Off. J. Int. Lact. Consult. Assoc..

