# Supplementary material for: An Exploration of Complementary Feeding Practices, Information Needs and Sources
Source: Int J Environ Res Public Health. 2019 Nov 6;16(22):4311. doi: 10.3390/ijerph16224311 (PMC6888072; doi:10.3390/ijerph16224311)
Supplement: Supplementary file 1 [file ijerph-16-04311-s001.pdf]

University of Glasgow | College of Medical, Veterinary & Life Sciences

An Exploration of Complementary Feeding Practices, Information Needs and Sources in North Lanarkshire

Thank you for completing this survey to find out how families are getting on with introducing solid foods. We will ask a few questions about your background, this information will be used confidentially and without linking it to your name. We will not share this with anybody else and if you don't wish to answer you don't have to.

First a few questions about yourself and your family:
What is your relationship to the child? *Mother -- Father – Other (specify)*: _______________
Is this your first baby? *Yes /No*.
If no, how many other children have you weaned before? __________

Your post code: ________________

Mother		Father	
Age: __________		Age: __________	
Mother's qualifications (tick all that apply):<br>Degree (or degree level qualification)<br>Scottish Highers or 'A' levels<br>Scottish standard grade or GCSE<br>No formal qualifications<br>Other qualifications (please state):<br>______________________	[ ]<br>[ ]<br>[ ]<br>[ ]<br>[ ]	Father's qualifications (tick all that apply):<br>Degree (or degree level qualification)<br>Scottish Highers or 'A' levels<br>Scottish standard grade or GCSE<br>No formal qualifications<br>Other qualifications (please state):<br>______________________	[ ]<br>[ ]<br>[ ]<br>[ ]<br>[ ]

Now a little information about your baby:
1. How old is your child now? *Age _____ months* (*If aged over 2 years conclude survey here)*
2. Is your baby a boy or a girl? *Male/ Female*
3. Has your baby had any major health problems since birth? *Yes / No*
   If yes, please describe them:

____

4. How much advice and help have you received about starting solids?
   A lot / Some / None / Not enough: if so what extra help or advice would you have liked?

____

5. If you received information, please indicate below how much you received: *(Please tick)*

	**None**	**Some**	**A lot**
First Fun Foods from NHS			
Other books or leaflets			
The internet			
- NHS			
- Forums (i.e. Netmums)			
- Baby food company webpages			
- Other			
Apps (Please specify): ______________________			
Social media groups, e.g. Instagram, Facebook, WhatsApp			
Your Health Visitor or a member of the HV team			
Your GP			
Family & friends			
Weaning groups			
Other support groups, e.g. breastfeeding/young mum/lone parent groups			

Infant Questions

6. What kind of information were you given? (Tick as many as apply)

Weaning starting age	[ ]
Types of foods to give	[ ]
Types of foods to avoid	[ ]
Nutrient information e.g. Iron, Calcium, Sugar, Salt, etc.	[ ]

7. Were you given any practical advice/ support?

How to cook baby foods	[ ]
How to store baby foods	[ ]
Economic benefits of cooking your own baby food	[ ]

8. What are/were your concerns regarding weaning?

What foods to give at different stages	[ ]
Texture of foods	[ ]
Safety of giving certain foods	[ ]
Number of milk feeds on top of solid foods	[ ]
Gagging	[ ]
Choking	[ ]
Food allergies	[ ]
Not giving enough food	[ ]
Baby not liking foods	[ ]
Giving treats, e.g. chocolate, sweets	[ ]
Knowing what is healthy	[ ]
Organic foods	[ ]
Processed foods	[ ]
Sweet foods/sugar	[ ]
Others (Please specify):	[ ]

____

9. What do you think would be useful for advice about weaning?

Fayres	[ ]
Small group sessions with food demonstrations	[ ]
Apps	[ ]
Social Media	[ ]
Videos:	[ ]
- With information on what food to give?	[ ]
- With information on how to feed?	[ ]
- With information on how to cook baby food?	[ ]
- With information on how to store home-made baby food?	[ ]
- With ideas of menus?	[ ]
- With information on portions?	[ ]
Other (please specify):	[ ]

____

In the next page, we want to know what foods your baby is eating

**1. Retrospective**	**Age in months**
Was your child breastfed? If yes, until what age? If not at all, put 0.	
What age was your child when you first introduced solid foods?	
**2. Drinks**	**Insert number into either:**
Does your child ever drink any of the following:<br><br>If yes, approximately how often? If not, put 0 times per day.	**Times per day**
Breast milk	
Formula milk	
Cow's milk	
Diluting juice, fruit juice, smoothies, fruit shoots or fizzy drinks	
Water as a drink	
Any other kind of milk such as soya or goat's milk? If yes, what kind?<br><br>____	
**3. Solid Feeding and Dietary Diversity**	
Does your child ever eat any of the following:<br><br>If yes, approximately how often? If not, put 0 times per week.	**Times per week**
Meat, fish or eggs? (including burgers, sausages, fish fingers etc., but not commercial baby foods)	
Commercial baby foods? (including jars, and savoury snacks, pouches, ready meals and any ready-made foods for babies)	
Green leafy vegetables? E.g. cauliflower, cabbage, kale, broccoli, Brussels sprouts, spinach or similar (not including ingredients in processed foods)	
Any solid, sour (sharp), uncooked fruits such as orange, tangerine, lemon, green apple (excluding juice, smoothies and processed foods)?	
Other fruits? E.g. banana, apple, pear	
Any sweet snacks, such as biscuits, cake, pastries, chocolate or dried fruit?	

**4. Do you give your infant vitamin supplements?**	**Yes/No**
If yes, please indicate which type. (Tick all boxes that apply)	
Healthy Start Vitamin Drops	
Abidec Multivitamin Drops	
Baby Ddrops Vitamin D	
Wellbaby Multi-vitamin liquid	
Wellbaby Vitamin D drops	
Wellbaby Multi-vitamin drops	
Others (please specify): ________________________	
Are you aware of the vitamin recommendations for your infant? If so, where did you receive this information? ________________________	Yes/ No
**5. On average, how many times per day does your child have:**	**Times per day**
Any meal or snack?	
Finger foods eaten by himself / herself?	
Spooned foods eaten by himself / herself?	
**6. Feeding behaviour**	
Are you finding it hard to get your child to eat solids?	Yes/No
Are there any foods, tastes or textures your child really doesn't like?	Yes/No
Generally, how is your child's appetite?<br>1 2 3 4 5 6<br>1 = very poor --- 6 = exceptionally good	

Thank you for completing this questionnaire!
